# Bayesian spatial modelling of malaria burden in two contrasted eco-epidemiological facies in Benin (West Africa): call for localized interventions

**DOI:** 10.1186/s12889-022-14032-9

**Published:** 2022-09-16

**Authors:** Barikissou Georgia Damien, Akoeugnigan Idelphonse Sode, Daniel Bocossa, Emmanuel Elanga-Ndille, Badirou Aguemon, Vincent Corbel, Marie-Claire Henry, Romain Lucas Glèlè Kakaï, Franck Remoué

**Affiliations:** 1 Centre de Recherche Entomologique de Cotonou, Ministère de la Santé, Cotonou, Benin; 2 Population Research Center (CEFORP), University of Abomey-Calavi, Cotonou, Bénin; 3 MIVEGEC (Maladies Infectieuses et Vecteurs: Ecologie, Génétique, Evolution Et Contrôle), University of Montpellier, IRD, CNRS, Montpellier, France; 4 Laboratoire de Biomathématiques et d’Estimations Forestières, Université d’Abomey-Calavi, Abomey-Calavi, Benin; 5 Present Address: Pôle des Technlogies de l’Information et de la Communication pour le Développement (ICT4D), IT4LIFE, Dakar, Sénégal; 6 Universités Paris 8, UFR Etudes – Recherche – et Ingénierie en territoires – Environnements – Société, Saint-Denis, France; 7 Departement of Medical Entomology, Centre for Research in Infectious Diseases (CRID), P.O. BOX 13591, Yaoundé, Cameroon; 8 Department of Animal Biology, Faculty of Science of the University of Dschang, Vector Borne Diseases Laboratory of the Biology and Applied Ecology Research Unit (VBID-URBEA), Dschang, Cameroon; 9 Departement de Santé Publique, Faculté des Sciences de la Santé, Université d’Abomey-Calavi, Cotonou, Benin

**Keywords:** Malaria, Risk mapping, *Plasmodium falciparum*, Decision-making, INLA

## Abstract

**Background:**

Despite a global decrease in malaria burden worldwide, malaria remains a major public health concern, especially in Benin children, the most vulnerable group. A better understanding of malaria’s spatial and age-dependent characteristics can help provide durable disease control and elimination. This study aimed to analyze the spatial distribution of *Plasmodium falciparum* malaria infection and disease among children under five years of age in Benin, West Africa.

**Methods:**

A cross-sectional epidemiological and clinical survey was conducted using parasitological examination and rapid diagnostic tests (RDT) in Benin. Interviews were done with 10,367 children from 72 villages across two health districts in Benin. The prevalence of infection and clinical cases was estimated according to age. A Bayesian spatial binomial model was used to estimate the prevalence of malaria infection, and clinical cases were adjusted for environmental and demographic covariates. It was implemented in R using Integrated Nested Laplace Approximations (INLA) and Stochastic Partial Differentiation Equations (SPDE) techniques.

**Results:**

The prevalence of *P. falciparum* infection was moderate in the south (34.6%) of Benin and high in the northern region (77.5%). In the south, the prevalence of *P. falciparum* infection and clinical malaria cases were similar according to age. In northern Benin children under six months of age were less frequently infected than children aged 6–11, 12–23, 24–60 months, (*p* < 0.0001) and had the lowest risk of malaria cases compared to the other age groups (6–12), (13–23) and (24–60): OR = 3.66 [2.21–6.05], OR = 3.66 [2.21–6.04], and OR = 2.83 [1.77–4.54] respectively (*p* < 0.0001). Spatial model prediction showed more heterogeneity in the south than in the north but a higher risk of malaria infection and clinical cases in the north than in the south.

**Conclusion:**

Integrated and periodic risk mapping of *Plasmodium falciparum* infection and clinical cases will make interventions more evidence-based by showing progress or a lack in malaria control.

**Supplementary Information:**

The online version contains supplementary material available at 10.1186/s12889-022-14032-9.

## Background

Between 2000 and 2019, malaria incidence rates in the World Health Organization (WHO) African Region reduced from 368 to 222 per 1000 population at risk, but increased to 232 in 2020 [1]. During this same period, malaria mortality rates decreased by 63%, from 150 to 56 per 100 000 population at risk, before rising to 62 in 2020 [1–3] before increasing. The use of Insecticide Treated Nets (ITNs) and Indoor residual Spraying (IRS) were considered to have made a major contribution to the reduction in malaria burden since 2000. ITNs was estimated to account for 50% of the decline in parasite prevalence among children aged 2–10 years in sub-Saharan Africa between 2001 and 2015 [4, 5]. However, indigenous malaria cases remain high in most African countries and need more attention and intervention [6]. Children under five years of age are particularly susceptible to malaria illness, infection and death [1].

Evaluating the impact of interventions is essential to designing more efficient and sustainable strategies for malaria control and elimination [7]. Frequent spatial and temporal mapping of malaria burden can be a valuable tool to measure progress in malaria control and elimination. The spatial modelling of malaria data can help the National Malaria Control Programme (NMCP) adjust the intervention.

Malaria is a major public health issue in Benin, especially among children under five years and pregnant women [8]. Malaria remains endemic, perennial in almost all regions, and seasonally dependent in the North [7, 9, 10]. High levels of *Anopheles* vectors resistance to insecticides were also described by many studies [11–13]. In 2015, malaria accounted for approximately 40% of care-seeking among the global population and 44.5% among children under five years old [14]. The National Malaria Control Programme (NMCP) of Benin was initiated in 1982. From 2006 to 2010, 2011 to 2018 and from 2017 to 2021, NMCP defined several strategies related to the intensification of malaria control and elimination, which were mainly based on the use of Long-lasting Insecticide Treated Nets (LLINs), indoor residual spraying (IRS), intermittent preventive treatment (IPTp-SP) with sulfadoxine-pyrimethamine, and prompt diagnosis and access to treatment with artemisinin-based combination therapy (ACT).

Nevertheless, the effect of malaria interventions across the eco-epidemiological facies remains poorly understood due to the absence of an active and rigorous surveillance system. In Benin, *Plasmodium falciparum* transmission shows seasonal patterns with an increase in the rainy season. The Demographic Health Survey (DHS) is carried out nationally every six years. It was conducted in the dry season, from November 6, 2017 to February 28, 2018, a period of low malaria incidence. This survey included 6156 individuals nationwide. The DHS is not solely dedicated to malaria but nevertheless gives an idea of the epidemiology of malaria in Benin. It confirms that the prevalence of malaria remains very high in the north of Benin (40% in the DHS in the dry season versus 77.5% in our study in the rainy season) and average in the south (23% in the DHS versus 34.6% in our study in the rainy season). This proves that the burden of malaria has not considerably decreased in Benin over the last ten years. This is why the team decided to share this data, which is still relevant and can motivate the repetition of this study and analysis design in other regions of Benin using the same approaches and motivating intervention, especially in the rainy season, the critical period of transmission.

After ten years of control, the current study aims to fill knowledge gaps on the spatial distribution of malaria infection in two different ecological settings using age range and geospatial modelling techniques. The relationship between the distribution of malaria vectors (or parasites) and environmental factors (e.g. temperature, rainfall, humidity, vegetation, proximity to waterways) has been well-established [15, 16]. Geostatistical models can estimate the environment-disease relation at known locations over a continuous space and predict malaria risk and uncertainty at locations where data on transmission is unavailable [15, 16]. These models also consider spatial dependence within the data by incorporating location-specific random effects since common exposures similarly influence disease transmission in neighbouring regions [17].

## Methods

### Study area

**Fig. 1:**
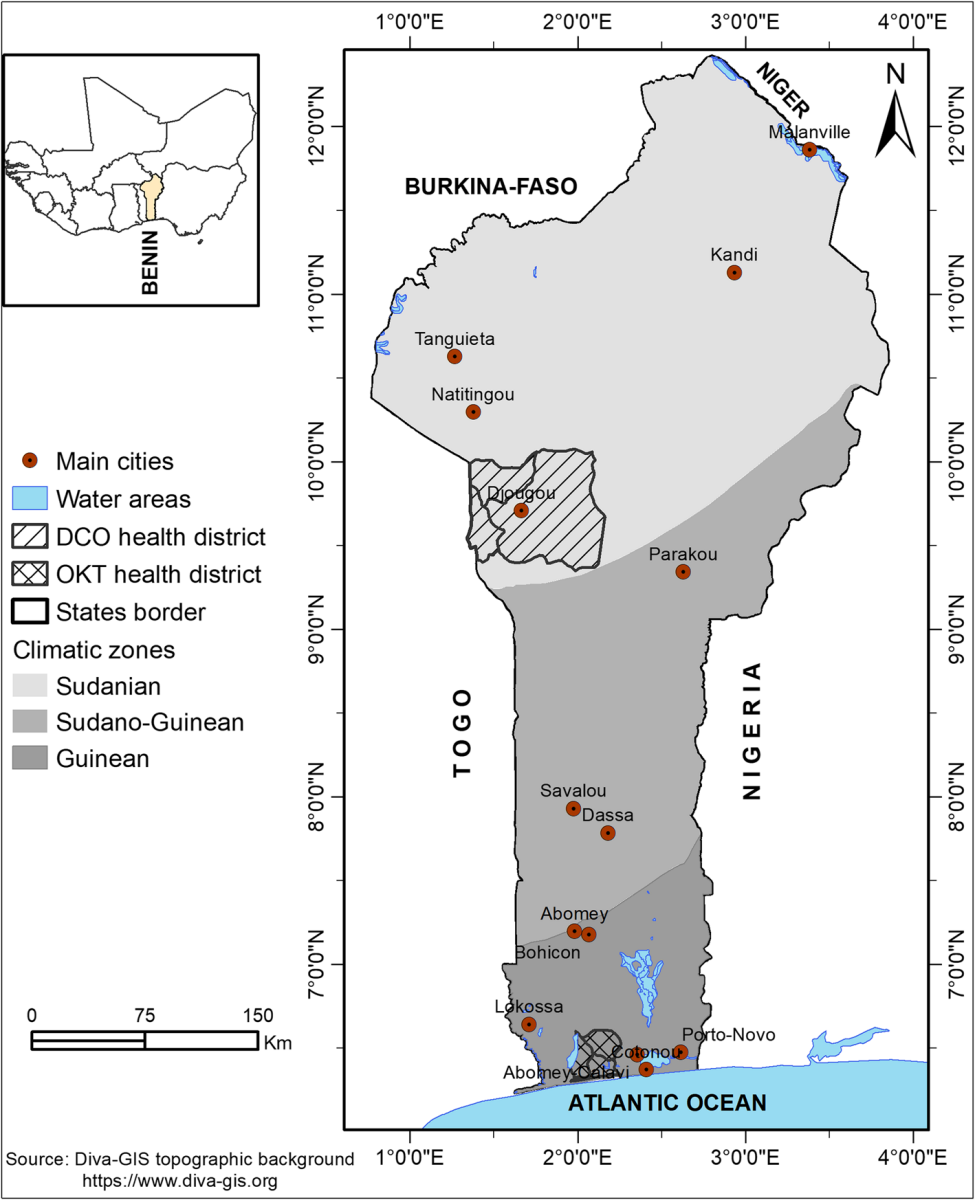
Map of the two study regions in Republic of Benin

The study was carried out in the Ouidah–Kpomassè–Tori Bossito (OKT) and Djougou–Copargo–Ouaké (DCO) health districts in Benin, West Africa in 2011, [18]. OKT is located in Southwestern Benin and 50 km from Cotonou (Fig. 1).

DCO is located in northern Benin and is at a 381 km distance from Cotonou (Fig. 1). The surface areas of OKT and DCO are 932 km^2^ and 5,505 km^2^, respectively. The commune of Ouidah, Kpomassè, Tori Bossito, Djougou, Ouaké and Copargo are located at 5 m, 27 m, 42 m, 421 m, 654 m and 396 m altitude respectively. Temperatures range between 22° C and 35° C, with an average of 27° C. In the south, the primary rainy season is from March to July; there is a short dry season from July to September and a short-wet season from mid-September to mid-November. Northern Benin only presents one rainy season (May to September, with most rain in August) and one hot, dry season.

A location map of the two study areas relative to the country was already elsewhere [18]. The OKT and DCO populations were 286,711 and 411,835, respectively, in 2013 [19]. Under-five years old were around 17% of the total population [20]. The characteristics of these areas have been previously described [7, 18]. The data from the 2020 Benin statistical health showed that malaria prevalence was 87% in the Donga department (DCO health zone) and 24% in the Atlantique department (OKT health zone), [21].

### Malaria control program implemented in the study area

#### Prevention

In both health zones, malaria prevention strategies were implemented by Non-Governmental Organizations working locally to promote child health. A behavior change communication strategy through social mobilization and home visits via various channels was used at the community level via messages mostly to women and young children. LLINs were distributed to all households, but the utilization rate for children under five years old remained low [18]. IRS was not achieved in both areas. The IPTp-SP for pregnant women was also promoted, but its use (first and second doses) was about 25%. Exclusive breastfeeding (EBF) for children under six months was also much lower than expected (16%) [20].

#### Diagnosis and treatment

In Benin, all clinical malaria cases were confirmed by RDT and microscopy where possible. At the time of the survey, the malaria case confirmation policy before treatment with ACT was not applied to community health workers (CHW). About 10% of febrile children used ACT in the south and 17% in the North [20]. Since 2014, malaria diagnosis and treatment have been made by the CHW and at the OKT and DCO health centres. However, care-seeking issued frequently financial, distance from health centers, transport and the repeated stock-out of Rapid Diagnostic Tests and Artemisinin-based Combination Therapy with CHWs and at health centres.

### Study design

Data were extracted from a cross-sectional survey cluster design during the rainy season as described elsewhere in 2016 [18, 22]. A two-stage random sampling technique was applied. The inclusion criterion for villages was a population size of 1000–1800 inhabitants. The target population was children aged 0–60 months, living in the selected villages, whose parents gave their informed consent. Thirty-one and 42 villages were randomly selected in the OKT and DCO health zones, respectively. Each village’s geographic coordinates were recorded using a global positioning system (GPS) provided by Benin’s Institute of National Geography (ING). In Benin's health pyramid, the local subdivision unit where health indicators are calculated before being aggregated at the regional and national level is the health zone. It is also at this level that decisions are taken to improve the health conditions of the population.

### Data collection

#### Parasitological and clinical data collection

Parasitological and clinical data were collected for two days in each village through a household survey. On the first day, three trained nurses assisted by three local village helpers visited the children in their households. The nurse examined and recorded data (age, sex, clinical and parasitological information) on every child while a physician supervised the fieldwork. The CareStart™ RDT used the detection of the histidine-rich protein-2 (HPR2) specific to *Plasmodium falciparum*. Malaria infection was defined as asymptomatic positive RDT. A clinical malaria case was defined as an association between high axillary temperature (> = 37.5 C) plus a positive RDT. Cross-check quality control was regularly done on a randomly selected sample representing 10% of RDT.

#### Environmental and demographic data collection and processing

To assess the effect of exposures on the malaria prevalence in the two targeted regions (OKT in Southern Benin and DCO in Northern Benin), climatic data (temperature and humidity variables) were collected from the *AFRICLIM* database, with a higher resolution of 30 arc-seconds (~ 1 km at the equator) [23]. These data were derived from the *Worldclim* baseline data interpolated across Africa. Environmental and demographic data (slope, elevation, distances to waterlines/coastlines and population density) were recorded from the *WorldPop* database [24, 25] with a spatial resolution of three arc-seconds (~ 100 m, at the equator) while the land cover covariate was recorded from Copernicus database [26] with a resolution of three arc-second (see the data sources and details in Table 1). All covariates were first processed with the Geographic Information System (GIS) software ArcGIS version 10.1 to match them to each region. Environmental and demographic data were resampled to the same resolution (~ 1 km) as climatic covariates using “*Spatial analysis tools*” of ArcGIS.

**Table 1:** Covariates used for modelling malaria prevalence in the study regions

**Covariates code**	**Signification**	**Resolution**	**Sources**
ben_ppp	Population density	3 arcseconds	Worldpop
bio1_w30s	Annual temperature	30 arcseconds	Africlim
bio12_wc30s	Annual rainfall	30 arcseconds	Africlim
bio4_wc30s	Temperature seasonality	30 arcseconds	Africlim
bio16_wc30s	Rainfall wettest quarter	30 arcseconds	Africlim
mimq_wc30s	Moisture index of moist quarter	30 arcseconds	Africlim
pet_wc30s	Potential evapotranspiration	30 arcseconds	Africlim
miaq_wc30s	Moisture index of arid quarter	30 arcseconds	Africlim
dst_coastline	Distance to coastline	3 arcseconds	Worldpop
dst_waterways	Distance to waterways	3 arcseconds	Worldpop
srtm_slope	Slope	3 arcseconds	Worldpop
Srtm_topo	Topography	3 arcseconds	Worldpop
landcover	Land cover	3 arcseconds	Copernicus

### Data analysis

#### Exploratory analysis

Data from the 2011 EVALUT project [18, 22], the prevalence of malaria infection and clinical cases were considered. The number of uncomplicated malaria clinical cases and the proportion of infection per the total population, have been used in this study. The difference in the prevalence of infection and clinical cases among age groups was tested using the Chi-Square test of goodness-of-fit. Mapping the observed prevalence of malaria infection and clinical cases was performed using the *ggplot2* library of R software [27].

#### Model specification

The prevalence of malaria infection and cases were estimated using clinical data aggregated at the community level (i.e. village). Let *Y*_*i*_ be the number of malaria infection (i.e. the number of individuals with positive blood test) or the number of cases (i.e. the number of individuals with malaria symptoms and with a positive blood test) within a selected village of location *s*_*i*_ (*i* = *1,…, n*) from a given study region. Let *N*_*i*_*t* denote the number of children tested within each village *s*_*i*_.

Let ***X***_*i*_*t* be a vector of *p* environmental and demographic covariates at the centroid of the village *i*. We assume that the disease counts *Y*_*i*_ follow a binomial distribution. Y_i_ ~ *Binomial* (*N*_*i*_* t, P*_*i*_), where *P*_*i*_ is the proportion of clinical cases or malaria infection in the population. The prevalence, *P*_*i*_ of malaria is assumed to be associated with exposures (environmental and population covariates) through a *logit* link such that: $logit\;\left(P_i\right)={X^{\prime}_i}\beta$, where *β* is a vector of regression coefficients to estimate from the data.

Random components were incorporated into the model to account for heterogeneity within the data (malaria clinical cases or infection prevalence) over a given study region to account for the effects of spatial autocorrelation between communities. Thus, spatially-structured random effects associated with spatial dependence between villages were modelled using a *Gaussian Random Field (GF)*, *U* (*s*), which has a multivariate normal distribution with null vector as mean and a covariance matrix, **Ʃ** [17, 28]: *U (s)* ~ *Gaussian* (***0***, **Ʃ**). This formulation is linked to the *Generalised linear spatial model (GLSM)* that is completely specified as below [29, 30]:

1 $$\begin{array}{c}{Y}_{i}|\beta ,{{X}_{i}},u\left({s}_{i}\right),\theta \sim Binomial\left({N}_{i},{P}_{i}\right),i=1,\dots ,n\\ logit\left({P}_{i}\right)={X^{\prime}_{i}}\beta +u\left({s}_{i}\right)+v\left({s}_{i}\right)\\ \begin{array}{c}u\left(s\right)\sim Gaussian\left(0,\Sigma \right)\\ {cov}[u\left({s}_{i}\right),u({s}_{j})]\propto {\sigma }_{u}^{2}\rho \left[\frac{\left|\left|{s}_{i}-{s}_{j}\right|\right|}{\phi }\right],\end{array}\end{array}$$

where $\phi$ is a scale parameter (i.e. the range) and ${\sigma }_{u}^{2}$ the variance (or the sill) of the process to be estimated from the data. The vector *v*(*s*) are community-specific random effects that account for the non-spatial variation or measurement error at each location (known as the *nugget effect* in geostatistics), while *θ* is a vector of all hyperparameters of random effects.

The popular covariance function assumed for *GF* in spatial statistics is the *Mátern covariance* which was shown to be the solution of a *Stochastic Partial Differential Equations (SPDE)* [31] and is defined as follows:

2 $$\mathrm{c}({s}_{i},{s}_{j},k)\propto {\sigma }_{s}^{2}(k\left|\left|{s}_{i}-{s}_{j}\right|\right|{)}^{v}{K}_{v}\left(k\left|\left|{s}_{i}-{s}_{j}\right|\right|\right),$$

where ${K}_{v}$ is the *Bessel function* of order $v>0,$ representing a smoothing parameter ($v=1$ in this case). The practical range $\phi$ is defined as $\sqrt{8v}/k$ and represents the distance at which the autocorrelation is low (i.e. close to 0.10).

The prior distribution is assigned to other model parameters to complete the hierarchical Bayesian spatial model defined in Eq. (1). An identically independent distributed (iid) Gaussian prior with zero mean and precision ${\tau }_{v}$ is assumed for the random vector *v,* while a Gaussian prior with large variance is assumed for regression coefficients *β*. We assigned a Gamma prior on hyperparameters ${\tau }_{u}=1/{\sigma }_{u}^{2}$_,_ and ${\tau }_{v}=1/{\sigma }_{v}^{2}$ using their default values on the log-scale. Though there are other extensions for *SPDE* to account for *non-stationarity* in the latent field [31], we assumed the spatial process *U(s)* to be *stationary* and *isotropic* for each study region, i.e. its statistical properties are invariant via translation and rotation [17].

#### Data analysis and model validation

Covariates values were extracted at observed locations and standardized to facilitate model stability. Correlation analysis was performed on pre-selected covariates to remove those showing strong correlation (|r|> 0.8) (see Supplementary file 1). Model selection was performed by running first a binomial regression model (i.e. GLMs with binomial family, see Supplementary file 2 and Supplementary file 3) on the disease counts and using the Akaike information criterion (AIC) to select the parsimonious model. For each response variable, full models (with all covariates) were calibrated. Variables that were not significant at the 10% threshold were removed while taking into account their similarity group (the inclusion in the model of two covariates belonging to the same group for a correlation coefficient greater than 0.80 in absolute value was avoided). t Covariates satisfying inclusion criteria were used to perform the Bayesian analysis [30]. The set of parsimoniously selected covariates associated with malaria prevalence is presented in Table 1. The deviance information criterion (DIC), the Bayesian counterpart of AIC, was used to select the parsimonious spatial Bayesian models. To assess the spatial autocorrelation within the data, we calculated Moran’s I from the residuals of the GLM models fitted to the observed data (infection and clinical cases) and tested its significance using 99 permutations. Moran’s I measures the similarity between data points as a function of the spatial lag distance, and its value is close to null in the absence of spatial autocorrelation [32]. All these descriptive analyses were performed using the R software version 3.6.

The spatial modelling process was performed within the Bayesian framework using *INLA-SPDE* techniques instead of the long-runs of Markov chain Monte Carlo (MCMC), which are computer-intensive in the case of hierarchical modelling [33]. All Bayesian analysis were performed using the R-INLA package. Moreover, we predicted the prevalence of malaria infection and cases from the selected model at grid locations of size approximately one km^2^ covering the whole extent of each region using a projector matrix to interpolate a functional of the random field (i.e. the posterior distribution of malaria prevalence computed at the mesh nodes). Standard deviation and Bayesian credible interval (BIC) of the prediction were also derived to assess the uncertainty associated with the estimates of disease prevalence [30].

## Results

### Population description and sources

A total of 10,367 children aged 0 to 60 months were included. In OKT, 4,348 children were recruited from 31 villages. The median age was 29 (1^st^qtle = 14; 3^rd^qtle = 45). In DCO, 6,019 children were included from 42 villages. The median age was 29 (1^st^qtle = 12; 3^rd^qtle = 46). The male /female ratio was 1:1 and 1.1:1.0 in OKT and DCO, respectively.

### Sources of infection

*P. falciparum and P. malariae* species were present in both areas. In OKT, among 198 positive thick films, 82.3% were positive with P. *falciparum*, 16.7% were positive with *P. malaria* and 0.5% co-infection with P. *falciparum* + *P. malaria* species*.* In DCO, among 331 positive thick films, 96.3% were positive with P. *falciparum*, 2.7% positive with *P. malaria* and 0.6% co-infection with *P. falciparum* + *P. malaria* species*.* The mean parasite density of *P. falciparum* in children was 1113 (CI95%). As *P. falciparum* was a dominant species, the following analysis focused on this species.

### *Plasmodium falciparum* infection and clinical cases according to age

#### OKT health district

**Fig. 2:**
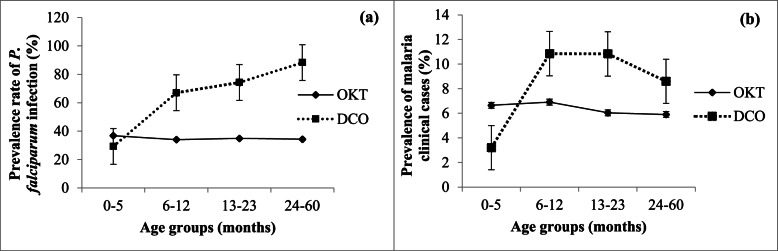
Prevalence rate of *Plasmodium falciparum* infection and prevalence rate of malaria clinical cases in OKT and DCO health districts—(**a**) represent the prevalence of *P. falciparum* according to the age groups, and (**b**) represent the prevalence of malaria clinical cases according to the age groups

The prevalence of *P. falciparum* infection was moderate in OKT 34.57% (1503/4348) (CI95% 33.17–35.99) and did not vary according to age, *p* = 0.8961, (Fig. 2a). The prevalence of *P. falciparum* infection among asymptomatic children was 28.65% (902/3148) [CI95% 27.10% – 30.26%].

Among 1,450 pathological episodes detected 457 were febrile (temperature >  = 37°5 C). A total of 267 (58.4%) clinical cases were confirmed with RDT and attributed to malaria (positive RDT plus signs). The prevalence of clinical malaria cases did not vary according to age group (*p* = 0.3918) (Fig. 2b).

#### DCO health district

The prevalence rate of *P. falciparum* infection was high in the north, 77.5% (4665/6019). Contrary to the south, the prevalence of infection increased with age in the DCO district (*p* < 0.0001). Children aged less than six months were less frequently infected than children aged 6–11, 12–23, 24–60 months OR = 4.92 [CI95% 3.75–6.45], OR = 6.99 [CI95% 5.33–9.17], and OR = 18.75 [CI95% 14.67–23.96] respectively (Fig. 2a). The proportion of *P. falciparum* infection in asymptomatic children was 64.03% (2348/3667) [CI95% 62.46%– 65.57%] in the DCO.

Among 1,770 pathological episodes identified at the health district level, 553 were febrile (temperature > 37°5 C). A total of 527 (95.3%) were attributed to malaria (RDT + signs). The prevalence of clinical malaria cases varied according to age groups. Children aged less than six months had a low risk of suffering from malaria compared to other age groups (6–12), (13–23) and (24–60): OR = 3.66 [CI95% 2.21–6.05], OR = 3.66 [CI95% 2.21–6.04], and OR = 2.83 [CI95% 1.77–4.54] respectively, *p* < 0.0001 (Fig. 2b).

### Mapping of malaria prevalence and clinical cases at observed locations and analysis of the spatial dependence

#### Mapping of the observed prevalence

**Fig. 3:**
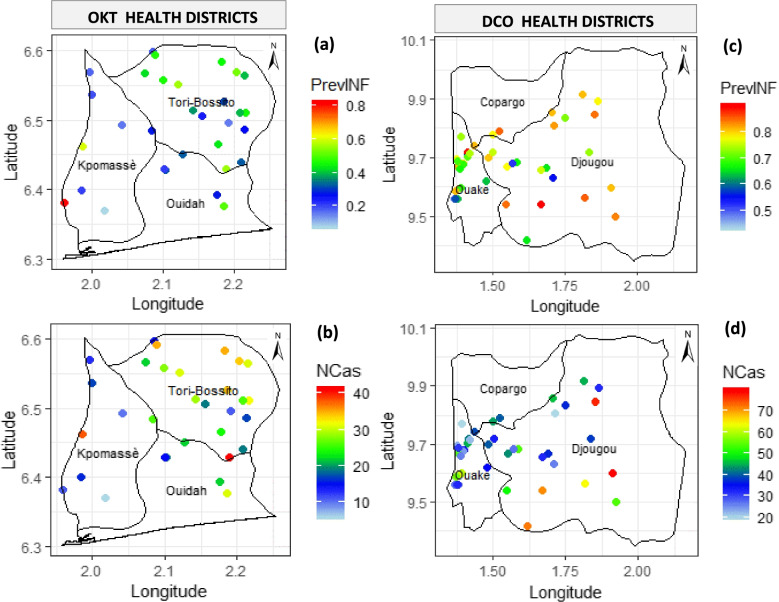
Raw maps of malaria prevalence at the observed locations—(**a**) and (**c**) prevalence of infection, (**b**) and (**d**) number of cases

From the raw maps of the malaria prevalence at the observed villages in the OKT region (Fig. 3), there is no location with unstable or very low transmission level (hypo-endemic areas), i.e. area with a prevalence of infection (PrevInf) less than 10%. Also, 77.41% of the sampled villages (24/31) have a prevalence of infection comprised between 10 and 50% (i.e. mesoendemic areas). About 16% (5/31) of the sampled villages are with hyperendemic transmission (50 < PrevInf < 75%), while only 3.22% of them are holoendemic (PrevInf > 75%). The number of malaria cases (Ncas) varied between 10 and 40, with a higher number in some villages located in the municipality of Kpomassè (Fig. 3a-b). However, the prevalence of infection is very high in the DCO region and varies between 45 and 90%. In contrast, malaria cases vary between 20 and 80, with the highest value in Djougou municipality (Fig. 3c-d).

#### Analysis of spatial dependence within the observed data

**Fig. 4:**
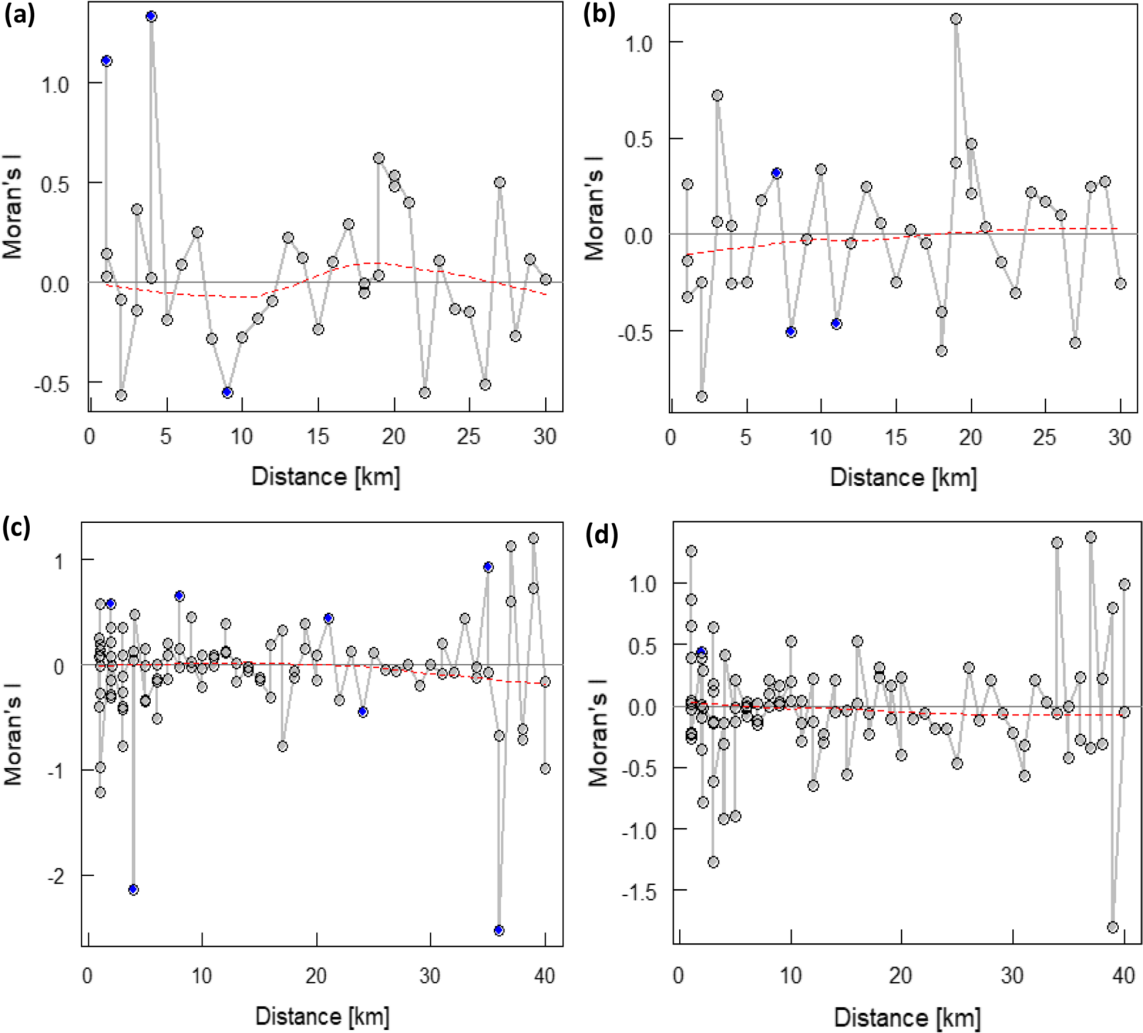
Correlograms for visualizing and testing the spatial autocorrelation within the observed data—(**a**) and (**b**) represent the plots of Moran’s I coefficients as function of distance for the prevalence of malaria infection and clinical cases, respectively in the OKT health district while (**c**) and (**d**) are Moran’s I plots as function of distance in the DCO health district. Blue color points represent the significant autocorrelation coefficients and the red line represents the overall trend of coefficients with distance

In the OKT health district, a clustering of observed data points (positive autocorrelation) around 1 km and 4.5 km and repulsion around 8.5 km (negative autocorrelation) for the prevalence of infection was noticed (Fig. 4a). For the observed clinical cases, there was clustering of data at a distance of around 6 km and repulsion at distances of 8 km and 11 km (Fig. 4b). Regarding the DCO health district, we noticed some significant autocorrelation coefficients showing both clustering and repulsion of the observed data at short distances. Clustering occurred at distances of 1 km, 5 km and 22 km, while repulsion occurred at 4 km, 25 km and 36 km for the prevalence of infection (Fig. 4c). The prevalence of clinical cases showed a significant clustering at 3 km (Fig. 4d).

### Prevalence of malaria with environmental and demographic exposures

The results of non-spatial and spatial models fitted to data revealed that the prevalence of malaria infection is affected by environmental and demographic exposures within the two regions (OKT and DCO). Exposures such as annual rainfall "bio12_wc30s", moisture index of the moist quarter “mimq_wc30s’’, distance to the coastlines “dst_coastlin”, topography “srtm_topo” and land cover influence significantly the prevalence of malaria infection within the OKT health district since the 95% credibility intervals of their mean estimates do not contain 0 **(**Table 2). These risk factors except the land cover showed a significant effect on malaria prevalence when considering the Bayesian binomial model without spatial component (Supplementary file 4 and Supplementary file 5). For the prevalence of malaria cases in the OKT region, moisture index of moist quarter “mimq_wc30s’’, moisture index of arid quarter and topography covariates were significant as all of their 95% credibility intervals do not contain 0.

**Table 2:** Results of Bayesian spatial binomial model fitted to the OKT data

**Sources of variation**	**Mean**	**SD**	**2.5%**	**50%**	**97.5%**
**Prevalence of infection**					
Intercept	-0.594	0.471	-1.550	-0.617	0.496
ben_ppp	0.198	0.223	-0.247	0.200	0.634
bio12_wc30s	-5.842	1.996	-9.771	-5.851	-1.865
bio4_wc30s	-0.202	0.174	-0.545	-0.202	0.142
mimq_wc30s	9.628	3.326	2.991	9.646	16.156
miaq_wc30s	-0.437	0.371	-1.182	-0.435	0.294
dst_coastlin	5.153	2.113	0.880	5.185	9.255
landcover	0.290	0.113	0.072	0.289	0.517
srtm_topo	0.916	0.321	0.276	0.918	1.548
***Hyperparameters***					
Theta1 for *U(s)*	-4.726	0.265	-5.205	-4.745	-4.16
Theta2 for *U(s)*	3.477	0.401	2.606	3.507	4.209
Precision ${\tau }_{v}$	18317.257	1.82E + 04	1260.762	12930.477	66416.689
**Prevalence of cases**					
Intercept	-1.624	0.231	-2.081	-1.643	-1.052
ben_ppp	0.141	0.120	-0.102	0.142	0.375
bio12_wc30s	-2.194	1.094	-4.325	-2.208	0.006
bio4_wc30s	-0.189	0.115	-0.414	-0.189	0.039
mimq_wc30s	3.982	1.793	0.385	4.001	7.487
miaq_wc30s	-0.405	0.186	-0.785	-0.401	-0.046
dst_coastlin	2.149	1.118	-0.106	2.165	4.325
landcover	0.126	0.076	-0.021	0.125	0.276
srtm_topo	0.539	0.177	0.184	0.541	0.885
***Hyperparameters***					
Theta1 for U(s)	-4.048	0.622	-5.263	-4.052	-2.812
Theta2 for U(s)	3.738	0.777	2.195	3.743	5.254
Precision ${\tau }_{v}$	19057.593	18750.000	1300.696	13531.915	68259.237

Regarding the DCO region, apart from the local mean prevalence (i.e. the intercept computed at the original scale), the population density "ben_ppp" is the only risk factor that significantly affects the prevalence of malaria infection. None covariate significantly affects the estimated prevalence of malaria cases since the 95% credibility interval of their effects contains 0 (Table 3). Contrarily to the OKT region, only GLM model showed significant effects of covariates on the estimates of malaria cases in DCO region (Supplementary file 3).

**Table 3:** Results of Bayesian spatial binomial model fitted to the DCO data

**Source of variation**	**Mean**	**SD**	**2.5%**	**50%**	**97.5%**
**Prevalence of infection**					
Intercept	1.043	1.240	-1.483	1.059	3.481
ben_ppp	-0.291	0.137	-0.560	-0.291	-0.017
bio12_wc30s	0.257	0.559	-0.870	0.264	1.342
bio16_wc30s	-0.476	0.682	-1.801	-0.484	0.900
dst_coastlin	0.318	0.432	-0.552	0.323	1.158
dst_waterway	0.180	0.137	-0.085	0.178	0.458
landcover	-0.073	0.098	-0.266	-0.073	0.119
pet_wc30s	-0.223	0.290	-0.784	-0.227	0.366
srtm_slope	0.062	0.090	-0.116	0.062	0.238
***Hyperparameters***					
Theta1 for *U(s)*	-2.557	1.742	-6.075	-2.515	0.785
Theta2 for *U(s)*	3.573	2.457	-0.974	3.450	8.703
Precision ${\tau }_{v}$	5.765	1.890	2.784	5.538	10.105
**Prevalence of cases**					
Intercept	-0.614	0.137	-0.873	-0.618	-0.330
ben_ppp	-0.016	0.170	-0.339	-0.022	0.336
bio4_wc30s	0.287	0.164	-0.035	0.285	0.615
bio12_wc30s	0.413	0.308	-0.192	0.411	1.027
dst_waterway	0.301	0.214	-0.120	0.299	0.729
miaq_wc30s	-0.122	0.154	-0.428	-0.122	0.181
mimq_wc30s	-0.272	0.321	-0.907	-0.272	0.361
srtm_slope	-0.031	0.119	-0.267	-0.032	0.205
srtm_topo	0.148	0.215	-0.282	0.150	0.570
landcover	0.132	0.130	-0.124	0.131	0.390
***Hyperparameters***					
Theta1 for *U(s)*	-5.452	2.164	-9.214	-5.638	-0.796
Theta2 for *U(s)*	4.814	0.971	2.769	4.878	6.559
Precision ${\tau }_{v}$	8.888	7.911	1.679	6.604	29.850

### Variation of the prevalence of malaria infection and clinical cases within the study regions

**Fig. 5:**
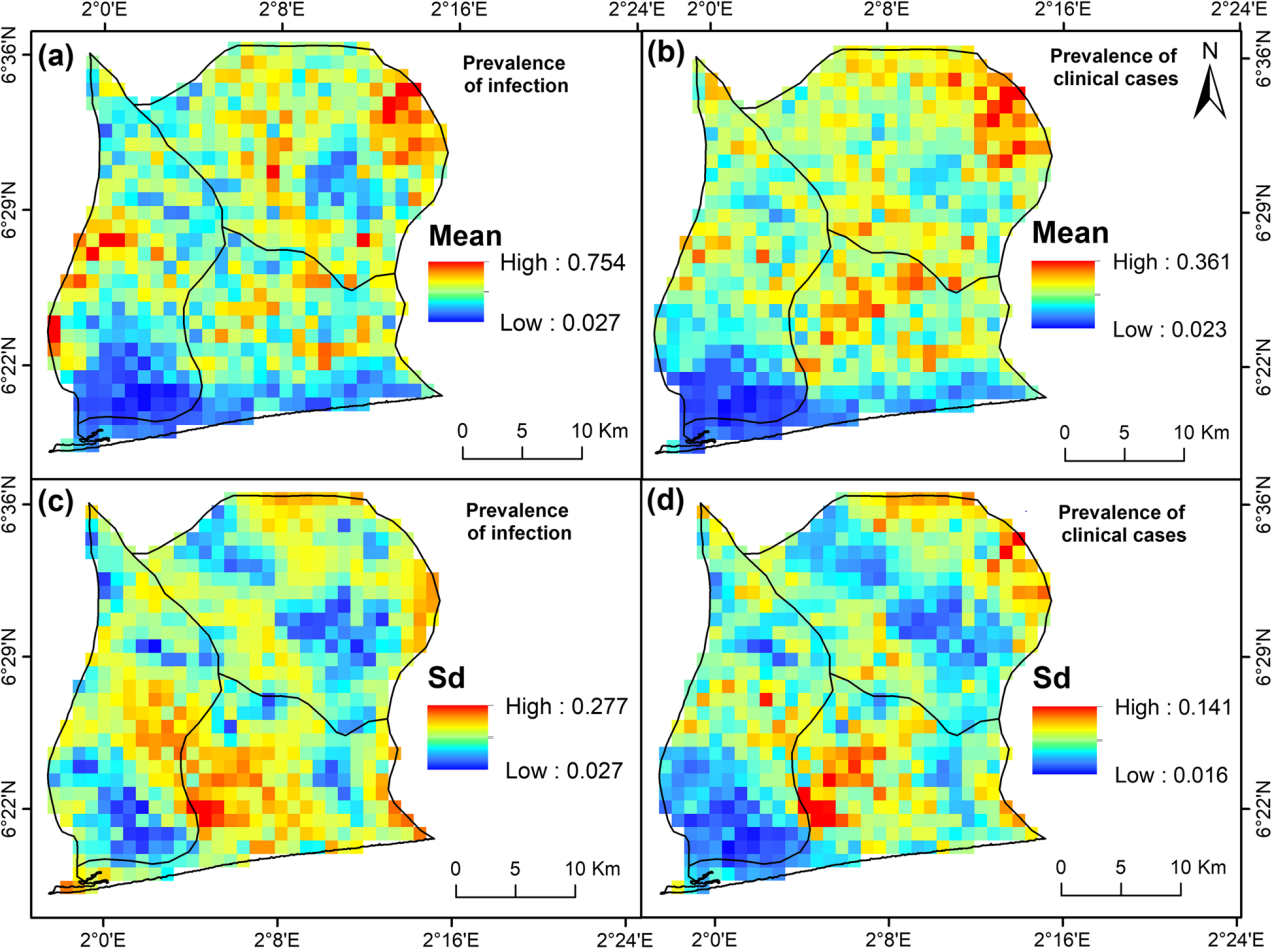
Mapping of the predicted prevalence of malaria within OKT (Ouidah—Kpomassè—Tori) health district—(**a**) and (**b**) mean posterior distribution of the estimates, (**c**) and (**d**) standard deviation of the estimates

**Fig. 6:**
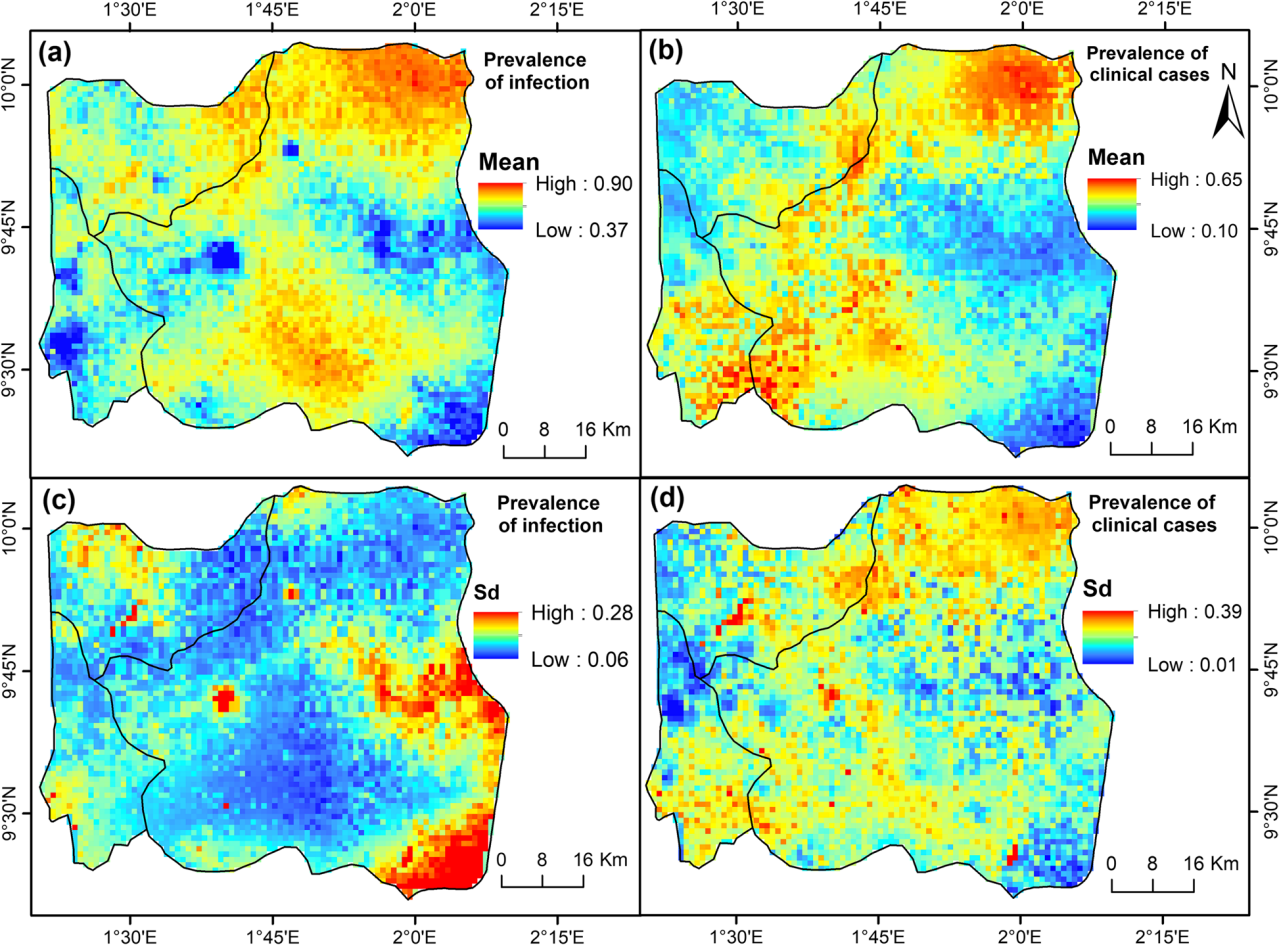
Mapping of the predicted prevalence of malaria within DCO (Djougou—Copargo—Ouaké) health district—(**a**) and (**b**) mean posterior distribution of the estimates, (**c**) and (**d**) standard deviation of the estimates

*Bayesian Generalised linear spatial models* implemented using stochastic partial differential equations (SPDE) combined with the INLA approach revealed that the prevalence of malaria infection varies locally within villages throughout the two study regions (Figs. 5 and 6). Malaria infection prevalence is predicted to be mesoendemicity or hyperendemicity (between 40 and 60%) in most locations in the study region, namely the villages within Ouidah and Tori Bossito communes. In comparison, a patch of holoendemicity (> 75%) is predicted to occur in the north-eastern part of Tori Bossito and the western part of Kpomassè (Fig. 5a). Globally, the malaria infection is expected to be low at the south of Kpomassè and Ouidah communes in the OKT health district, but with a higher standard deviation of about 20 to 28% especially in Ouidah (Fig. 5c). Regarding the prediction of the prevalence of malaria cases, moderate prevalence values occurred within the three communes of the OKT health district, with more importance in Tori Bossito than Ouidah and Kpomassè (Fig. 5b). A relatively low standard deviation of the estimates (1.6 to 14.0%) has been observed for the prevalence of clinical cases in the region with the highest values in Ouidah (Fig. 5d).

A contrarily, in the DCO region, a very high level of malaria infection was observed throughout the study area. Some areas located in the central and northern part of Djougou and Copargo communes had an estimated prevalence between 60 and 75% (i.e. hyperendemic areas), and others (extreme north of Djougou) had an estimated prevalence greater than 75% (i.e. holoendemic areas). However, some areas located at Ouaké and the south-eastern part of Djougou in the DCO health district has a mesoendemic transmission of malaria infection (10 to 50%) (Fig. 6a). The predicted prevalence of malaria cases was relatively high (40 to 65%) in some locations of the DCO health district, especially in the extreme north of Djougou (Fig. 6b). Moreover, the standard deviations were relatively low for the estimates of malaria infection (6% < SD < 28%), while they were relatively high for the calculations of malaria cases (1% < SD < 39%) (Fig. 6c-d).

## Discussion

Half part of care-seeking is attributable to malaria in Benin despite the high intervention packages implemented throughout the country. Routine data exist and help decision making but seem insufficient. Spatial modelling can help to observe disease indicators from new angles, allow for finer analysis and thus help to make more relevant decisions. The main objectives of this study were to address the spatial distribution of *P. falciparum* infection prevalence and clinical cases in children under five years old and to identify the importance of malaria burden in earlier infant age groups between two different ecological settings: southern Benin (two rainy seasons) and northern Benin (one rainy season).

### Spatial epidemiology as decision making tool

WHO recommended ensuring access to malaria prevention, diagnosis and treatment as part of universal health coverage by determining the most effective mix of interventions according to the local context and needs. This induce data collection or using existing data that reflect a need for subnational level [34]. Recent reforms are being introduced in the health department in Benin in order to reduce the burden of AIDS, Tuberculosis, Malaria, Hepatitis, Sexual Transmitted Infections and epidemic-prone diseases. Their effects are not yet measurable.

This is the first-time spatial analysis of malaria infection and morbidity focused on the OKT and DCO health districts. The two ecological regions do not have the same epidemiological profile. The OKT district was a mesoendemic and/or hyperendemic area with a perennial transmission [7]. Heterogeneity of *P. falciparum* infection has already been described in the OKT district in a previous study and was not surprising [7, 35]. A contrarily, the prevalence rate of *P. falciparum* was very high in northern Benin. The DCO district remained a hyperendemic area. The Malaria Atlas Project confirmed a similar pattern in the spatial epidemiology of *P. falciparum* [36, 37]. In Côte d’Ivoire, a West African country, a higher prevalence rate of *P. falciparum* infection was also predicted in the north compared to the south [38]. Vectors density, coverage in vector control tools, drugs, immunity, human genetics, social, demographic, and environmental factors could explain the difference in *P. falciparum* prevalence between northern and southern regions. National malaria programmes should determine the appropriate package of interventions for local level considering transmission intensity, as well as a good understanding of each area’s ecological, epidemiological, and social features. Interventions should be adapted and tailored to specific geographical areas within each country [39]. The lack of qualified human resources in data modelling was a huge obstacle in decision making. Challenges remain in convincing national institutions to actively participate and provide financial support.

### Constraints of malaria control

A 2010 study conducted at the national level in Benin showed that 34.5% of households in the north versus 28.3% in the south owned at least two LLINs. In opposite, 55.9% of children under five years slept under the LLINs the night before in the north compared to 68.1% in the south [40]. This coverage of vector control tools remains poor and can explain the high prevalence of malaria infection and clinical cases found.

The vector resistance to insecticide was high in all the studied health districts. *Anopheles gambiae*, the main malaria vector, is highly resistant to the standard insecticides [9, 13]. Data collected during the study showed that use of an LLIN was low (17–57%) and gave only 40 to 50% effectiveness against malaria clinical cases [18].

During the 2011 and subsequent campaigns, community awareness of LLIN use was mainly addressed to mothers, pregnant women and children. The understanding or perception induced by the communication campaigns is that the LLINs is to be used by women of childbearing potential and the children with whom they usually sleep. A large part of children was kept out from intervention by the messages delivered during awareness campaigns. The malaria awareness campaigns should therefore be reviewed in order to improve LLINs use rates among elder children and adult males.

In Benin, malaria control tools (LLINs, ACT, SP etc.) are available at the health facilities. The index of availability of malaria control tracers among health facilities offering this service provided by SARA survey was 82% in Donga department (DCO health zone), compared to 71% in Atlantique department (OKT health zone). However, stock-out of anti-malarial drugs were often noticed in certain health centres. Care-seeking at health centres and utilization of recommended treatment of malaria remains a challenge. One major reason is to be self-medication by herbal tea and other non-recommended drugs such as chloroquine, quinine and paracetamol [41].

### Age effect

Studies suggest a protective effect of maternal breastmilk against *P. falciparum* [42, 43]. So, in theory, children under six months of age are supposed to be protected against infection compared to the elder children due to EBF [44]. In the current study, in the south OKT district, the infection prevalence was similar among the four age groups. Children under six months of age (supposed to be exclusive to breastfed) were similarly infected than the oldest children. The same observation was seen among clinical cases. In the north, the prevalence of *P. falciparum* infection and the proportion of clinical patients were higher than in the south. The prevalence rate of infection was lower among children under six months of age than in the oldest. According to knowledge, the prevalence of parasitemia increases sharply beginning at about 20 weeks of age [44].

Nevertheless, children remain extraordinarily resistant to high parasitemia, fever, and severe malaria until about six months of age. This protection is associated with maternal immunoglobulin G (IgG) antibodies specific to *Plasmodium* antigens, as IgG levels decrease from birth over the first year of life. Another assumption is that the protection of infants may be associated with parasite growth-inhibitory factors such as lactoferrin and secretory IgA found in breast milk and maternal and infant sera [44, 45]. Further, data from the Democratic Republic of the Congo showed that EBF reduced the risk of clinical malaria in children under six months of age [43]. However, the assumption that maternal antibodies against malaria antigens were the basis of this protection was contradicted by at least one study [46]. We did not verify this association in the present study. However, in the DHS data from Benin, children under five years received EBF or breastfeeding accompanied by water and/or other liquids (without milk) for 4.5 months in the DCO area and only 3.0 months in OKT [20]. Thus, breastfeeding seems less important in the south than in the north. After the age of peak of the prevalence rate of infection, the number of clinical attacks of malaria per year dramatically declines. For example, in the DCO district, the prevalence of *P. falciparum* clinical cases decreased after 23 months, suggesting the beginning of immunological premonition acquisition [47, 48]. Then, from 23 to 60 months, the frequency of clinical cases diminishes. The age of onset of this protection is somewhat earlier with heavier transmission [42, 43], but protection rarely occurs before the age of two years [44]. In southern Benin, the prevalence rate of infection did not vary according to age group, probably due to the moderate level of malaria endemicity [7, 18]. To fully understand the link between EBF and malaria infection and disease burden, more studies should be carried out in Benin.

### Limitations

This study assessed the spatial heterogeneity of malaria risk in two ecological regions in Benin by using 2011 database. It estimated the disease prevalence adjusted for environmental and population covariates and found a significant relationship between temperature seasonality, rainfall, population density, moisture index and distance to the nearest waterways. However, the data were aggregated at village location in place of household. When GPS coordinates are available at the household level, similar models could be implemented to improve the smoothness of malaria risk prediction in the study regions. Considering household or individual-level data can allow the inclusion of other socio-demographic or personal characteristics (e.g. age, sex, access to a mosquito bed net, etc.) that are likely to influence disease transmission but not easy to obtain for aggregated data collected in village or county-level. The climate changes and the variations of temperature and precipitations change significantly with a cycle of 30 years average. However, mayor events like catastrophes and crisis can affect diseases epidemiology. Benin country did not experience this kind of event the last decade. Finally, more complex models could be implemented by assuming a non-stationary spatial process to evaluate its influence on models' outcomes [29].

## Conclusion

The spatial analysis concerns malaria infection and disease distribution among children under five years across two endemic and various areas in Benin. A higher risk of malaria infection and clinical cases were found in the north than in the south. The surveillance by geospatial approach and capacity building in modelling needs to be systematic and periodic to facilitate decision-making on the cost-effectiveness and global impact of malaria control efforts.

## Supplementary Information

**Additional file 1: Figure A1.** Correlation between preselected covariates (c & d) and their similarity groups (a & b) for the OKT region (top panel) and DCO region (bottom panel) – Cells with crosses are correlation coefficients that are not statistically significant.

**Additional file 2: Table A1.** Simple binomial GLM fitted to the OKT data.

**Additional file 3: Table A2.** Simple binomial GLM fitted to the DCO data.

**Additional file 4: Table A3.** Bayesian binomial model with random noises fitted to the OKT data

**Additional file 5: Table A4.** Bayesian binomial model with random noises fitted to the DCO data

## Data Availability

All relevant data supporting the conclusion of this article can be provided as an additional file.

## Abbreviations

ACT: Artemisinin-based combination therapy
CHW: Community Health Worker
DCO: Djougou–Copargo–Ouaké
DHS: Demographic Health Survey
GIS: Geographic Information System
GPS: Global positioning system
EBF: Exclusive breastfeeding
HPR2: Histidine-rich protein-2
ING: Institute of National Geography of Benin
INLA: Integrated Nested Laplace Approximations
IPTp-SP: Intermittent preventive treatment with sulfadoxine-pyrimétamine
IRS: Indoor residual spraying
LLINs: Long-lasting insecticide-treated nets
NMCP: National Malaria Control Programme
OKT: Ouidah–Kpomassè–Tori Bossito
RDT: Rapid diagnosis tests
SPDE: Stochastic Partial Differentiation Equations
WHO: World Health Organization
