# Supplementary material for: Bayesian spatial modelling of malaria burden in two contrasted eco-epidemiological facies in Benin (West Africa): call for localized interventions
Source: BMC Public Health. 2022 Sep 16;22:1754. doi: 10.1186/s12889-022-14032-9 (PMC9479262; doi:10.1186/s12889-022-14032-9)
Supplement: Supplementary file 1 — Additional file 1: Figure A1. Correlation between preselected covariates (c & d) and their similarity groups (a & b) for the OKT region (top panel) and DCO region (bottom panel) – Cells with crosses are correlation coefficients that are not statistically significant. [file 12889_2022_14032_MOESM1_ESM.pdf]

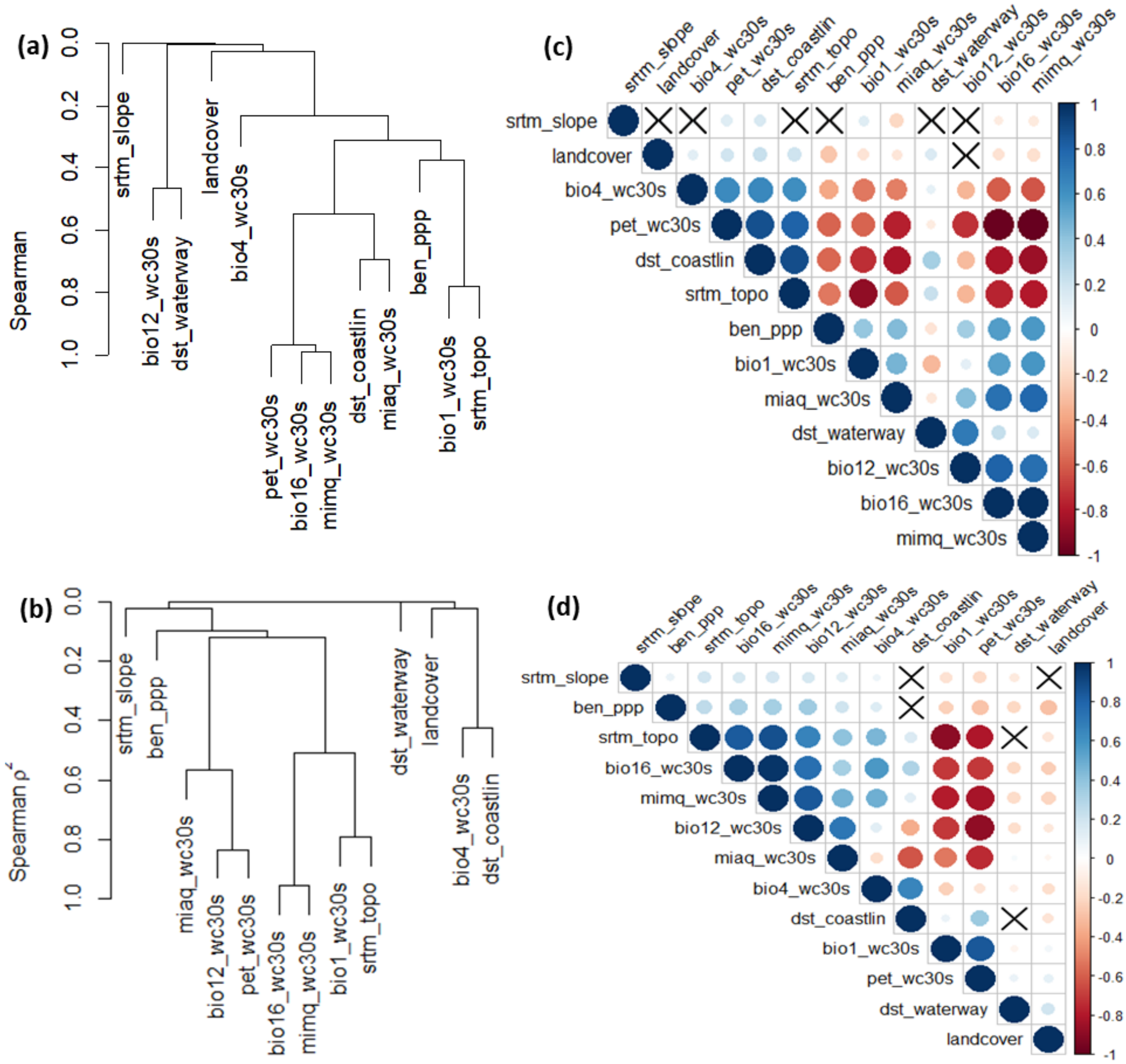

Figure A1. Correlation between preselected covariates (c & d) and their similarity groups (a & b) for the OKT region (top panel) and DCO region (bottom panel) – *Cells with crosses are correlation coefficients that are not statistically significant*
