# Supplementary material for: Bayesian spatial modelling of malaria burden in two contrasted eco-epidemiological facies in Benin (West Africa): call for localized interventions
Source: BMC Public Health. 2022 Sep 16;22:1754. doi: 10.1186/s12889-022-14032-9 (PMC9479262; doi:10.1186/s12889-022-14032-9)
Supplement: Supplementary file 2 — Additional file 2: Table A1. Simple binomial GLM fitted to the OKT data. [file 12889_2022_14032_MOESM2_ESM.pdf]

Table A1: Simple binomial GLM fitted to the OKT data

Source of variation	Estimate	Std. Error	z value	Pr(>|z|)
**Prevalence of infection**				
(Intercept)	-0.603	0.033	-18.303	< 2e-16
ben_ppp	0.117	0.052	2.267	0.023
bio1_wc30s	0.188	0.110	1.706	0.088
bio12_wc30s	-5.017	0.740	-6.781	< 0.001
bio16_wc30s	-3.347	1.210	-2.765	0.006
bio4_wc30s	-0.117	0.069	-1.697	0.090
dst_coastlin	5.156	0.710	7.260	< 0.001
landcover	0.202	0.039	5.214	< 0.001
miaq_wc30s	-0.266	0.086	-3.090	0.002
mimq_wc30s	10.874	1.267	8.584	< 0.001
pet_wc30s	-1.407	0.596	-2.361	0.018
srtm_topo	1.011	0.149	6.792	0.000
**Prevalence of cases**				
(Intercept)	-1.637	0.042	-38.689	< 0.001
ben_ppp	0.158	0.063	2.522	0.012
bio12_wc30s	-2.171	0.633	-3.433	0.001
bio4_wc30s	-0.201	0.071	-2.826	0.005
dst_coastlin	2.160	0.626	3.450	0.001
landcover	0.096	0.051	1.889	0.059
miaq_wc30s	-0.342	0.093	-3.688	< 0.001
mimq_wc30s	3.982	1.029	3.868	< 0.001
srtm_slope	0.083	0.050	1.665	0.096
srtm_topo	0.564	0.106	5.326	< 0.001

The table A1 showed the estimates of the binomial GLM fitted to the data from the OKT (Ouidah – Kpomassè – Tori) health district, where only covariates having a significant effect on *P. Falciparum* infection at 10% have been presented. Annual rainfall (bio12_wc30s), rainfall of wettest quarter (bio16_wc30s), evapotranspiration (pet_wc30s**)**, and moisture index of arid quarter (miaq_wc30s) have a negative effect with malaria infection, while population density (ben_ppp), distance to coastline (dst_coastlin), topography (srtm_topo), moisture index of moist quarter (mimq_wc30s) and land cover have a positive effect. Regarding the malaria clinical cases, annual rainfall, temperature seasonality, and moisture index of arid quarter showed a negative link with the observed response, while population density, distance to coastline, topography and moisture index of moist quarter revealed a positive link.
