# Supplementary material for: Bayesian spatial modelling of malaria burden in two contrasted eco-epidemiological facies in Benin (West Africa): call for localized interventions
Source: BMC Public Health. 2022 Sep 16;22:1754. doi: 10.1186/s12889-022-14032-9 (PMC9479262; doi:10.1186/s12889-022-14032-9)
Supplement: Supplementary file 3 — Additional file 3: Table A2. Simple binomial GLM fitted to the DCO data. [file 12889_2022_14032_MOESM3_ESM.pdf]

Table A2: Simple binomial GLM fitted to the DCO data

Source of variation	Estimate	Std. Error	z value	Pr(>|z|)
**Prevalence of infection**				
(Intercept)	1.082	0.032	33.335	< 2e-16
ben_ppp	-0.370	0.056	-6.664	2.66E-11
bio12_wc30s	0.553	0.207	2.669	0.008
bio16_wc30s	-0.775	0.263	-2.949	0.003
dst_coastlin	0.516	0.163	3.178	0.001
dst_waterway	0.167	0.058	2.863	0.004
landcover	-0.058	0.041	-1.394	0.163
pet_wc30s	-0.313	0.115	-2.711	0.007
srtm_slope	0.093	0.038	2.470	0.014
**Prevalence of cases**				
(Intercept)	-0.759	0.030	-25.153	< 2e-16
ben_ppp	-0.093	0.042	-2.197	0.028
bio12_wc30s	0.469	0.107	4.399	1.09E-05
bio4_wc30s	0.217	0.049	4.382	1.18E-05
dst_waterway	0.214	0.061	3.490	0.000
landcover	0.131	0.041	3.220	0.001
miaq_wc30s	-0.091	0.047	-1.934	0.053
mimq_wc30s	-0.331	0.102	-3.255	0.001
pet_wc30s	0.211	0.106	1.998	0.046
srtm_slope	-0.061	0.037	-1.649	0.099
srtm_topo	0.281	0.094	2.986	0.003

The table A2 showed the estimates of the binomial GLM fitted to the data from the DCO (Djougou – Copargo – Ouaké) health district, where only exposures having a significant effect on *P. Falciparum* infection at 10% have been presented. Population density, rainfall of wettest quarter and evapotranspiration are negatively linked to malaria infection while annual rainfall, distances to coastlines and waterways and slope are positively linked to malaria infection. For the malaria clinical cases, population density, moisture index of arid quarter, moisture index of moist quarter and slope showed a negative relationship with the observed response, while annual rainfall, temperature seasonality, distances to waterways, land cover, evapotranspiration and topography depicted a positive link with the observed outcome.
