# Supplementary material for: Bayesian spatial modelling of malaria burden in two contrasted eco-epidemiological facies in Benin (West Africa): call for localized interventions
Source: BMC Public Health. 2022 Sep 16;22:1754. doi: 10.1186/s12889-022-14032-9 (PMC9479262; doi:10.1186/s12889-022-14032-9)
Supplement: Supplementary file 4 — Additional file 4: Table A3. Bayesian binomial model with random noises fitted to the OKT data [file 12889_2022_14032_MOESM4_ESM.pdf]

Table A3: Bayesian binomial model with random noises fitted to the OKT data

Sources of variation	**Mean**	**SD**	**2.5%**	**50%**	**97.5%**
**Prevalence of infection**					
(Intercept)	-0.654	0.130	-0.913	-0.654	-0.398
ben_ppp	0.154	0.208	-0.257	0.153	0.567
bio12_wc30s	-5.812	2.761	-11.292	-5.810	-0.354
bio16_wc30s	-2.398	4.760	-11.788	-2.413	7.063
bio4_wc30s	-0.214	0.262	-0.734	-0.213	0.302
dst_coastlin	5.921	2.677	0.620	5.919	11.225
landcover	0.211	0.156	-0.098	0.211	0.520
miaq_wc30s	-0.306	0.356	-1.013	-0.305	0.397
mimq_wc30s	11.301	4.898	1.561	11.312	20.970
pet_wc30s	-1.232	2.196	-5.583	-1.233	3.117
srtm_topo	0.840	0.313	0.221	0.840	1.462
Precision τ_v	2.310	0.791	1.074	2.205	4.143
**Prevalence of cases**					
(Intercept)	-1.650	0.054	-1.764	-1.648	-1.548
ben_ppp	0.165	0.081	0.008	0.164	0.333
bio12_wc30s	-2.132	0.791	-3.691	-2.138	-0.533
bio4_wc30s	-0.205	0.091	-0.392	-0.203	-0.026
dst_coastlin	2.131	0.784	0.556	2.134	3.683
landcover	0.102	0.067	-0.026	0.101	0.241
miaq_wc30s	-0.357	0.119	-0.606	-0.354	-0.129
mimq_wc30s	3.927	1.293	1.318	3.935	6.479
srtm_slope	0.085	0.064	-0.042	0.085	0.214
srtm_topo	0.563	0.131	0.303	0.563	0.822
Precision τ_v	9714.100	17023.120	6.699	322.760	59127.390

Table A3 showed the estimates of the effects of malaria exposures in the OKT health district using Bayesian GLMM (generalized linear mixed models) without spatial component. Only annual rainfall showed a significant negative link with the risk of infection by *P. falciparum* when including village-specific random effects in the model. In contrarily, distance to coastline, moisture index of moist quarter and topography revealed a significant positive link with malaria infection. Regarding the malaria clinical cases, the trends of exposures are exactly similar to those observed with frequentist GLMs in Table A1. However, there is a low variability within the observed malaria clinical cases (high precision) as compared to malaria infection outcomes.
