# Supplementary material for: Bayesian spatial modelling of malaria burden in two contrasted eco-epidemiological facies in Benin (West Africa): call for localized interventions
Source: BMC Public Health. 2022 Sep 16;22:1754. doi: 10.1186/s12889-022-14032-9 (PMC9479262; doi:10.1186/s12889-022-14032-9)
Supplement: Supplementary file 5 — Additional file 5: Table A4. Bayesian binomial model with random noises fitted to the DCO data [file 12889_2022_14032_MOESM5_ESM.pdf]

**Table A4**: Bayesian binomial model with random noises fitted to the DCO data

| Sources of variation | **Mean** | **SD** | **2.5%** | **50%** | **97.5%** |
|---|---|---|---|---|---|
| Prevalence of infection | | | | | |
| (Intercept) | 1.076 | 0.076 | 0.925 | 1.076 | 1.226 |
| ben_ppp | -0.286 | 0.136 | -0.553 | -0.287 | -0.015 |
| bio12_wc30s | 0.333 | 0.522 | -0.703 | 0.335 | 1.359 |
| bio16_wc30s | -0.578 | 0.645 | -1.848 | -0.579 | 0.700 |
| dst_coastlin | 0.380 | 0.408 | -0.429 | 0.381 | 1.183 |
| dst_waterway | 0.158 | 0.127 | -0.093 | 0.158 | 0.410 |
| landcover | -0.075 | 0.096 | -0.265 | -0.075 | 0.113 |
| pet_wc30s | -0.275 | 0.271 | -0.809 | -0.275 | 0.260 |
| srtm_slope | 0.065 | 0.089 | -0.110 | 0.065 | 0.239 |
| Precision $\tau_v$ | 5.682 | 1.835 | 2.888 | 5.415 | 10.020 |
| Prevalence of cases | | | | | |
| (Intercept) | -0.671 | 0.101 | -0.868 | -0.671 | -0.471 |
| ben_ppp | -0.113 | 0.136 | -0.383 | -0.113 | 0.155 |
| bio4_wc30s | 0.257 | 0.163 | -0.064 | 0.256 | 0.579 |
| bio12_wc30s | 0.441 | 0.278 | -0.107 | 0.440 | 0.990 |
| dst_waterway | 0.300 | 0.198 | -0.089 | 0.299 | 0.692 |
| miaq_wc30s | -0.125 | 0.149 | -0.419 | -0.125 | 0.168 |
| mimq_wc30s | -0.298 | 0.319 | -0.929 | -0.298 | 0.333 |
| srtm_slope | -0.015 | 0.115 | -0.242 | -0.015 | 0.213 |
| srtm_topo | 0.195 | 0.198 | -0.196 | 0.195 | 0.587 |
| landcover | 0.126 | 0.129 | -0.129 | 0.126 | 0.380 |
| Precision $\tau_v$ | 2.841 | 0.804 | 1.538 | 2.749 | 4.671 |

Table A4 showed the estimates of the effects of malaria determinants in the DCO health district using Bayesian GLMM (generalized linear mixed models) without spatial component. In the presence of village-specific random effects, only the population density showed a significant effect on the malaria infection. No covariate has significant effect on the malaria clinical cases in the DCO region since the credibility interval of all estimates contain zero.
